# Assessment and Management of Obesity and Self-Maintenance (AMOS): An Evaluation of a Rural, Regional Multidisciplinary Program

**DOI:** 10.3390/ijerph191912894

**Published:** 2022-10-08

**Authors:** Sarah J. Prior, Sharon P. Luccisano, Michelle L. Kilpatrick, Giuliana O. Murfet

**Affiliations:** 1 Tasmanian School of Medicine, University of Tasmania, Burnie, TAS 7320, Australia; 2 Diabetes Centre, Tasmanian Health Service—North West, Burnie, TAS 7320, Australia; 3 Menzies Institute for Medical Research, University of Tasmania, Hobart, TAS 7000, Australia; 4 School of Public Health, University of Technology Sydney, Ultimo, NSW 2007, Australia

**Keywords:** obesity care model, nurse practitioner, allied health, motivation for weight loss, barriers to obesity management, diabetes management

## Abstract

Obesity is common in rural areas, and reduced specialist healthcare access impedes its management. A pilot nurse-practitioner-led Assessment and Management of Obesity and Self-Maintenance (AMOS) Clinic focused on individualised obesity care in people living with type 2 diabetes delivered in a rural setting. This study aimed to explore participant and staff experiences of the multidisciplinary obesity clinic to identify barriers and facilitators to self-care, health, and well-being. A two-stage, mixed-method design was used. Initially, three focus groups involving a sample of AMOS participants and semi-structured staff interviews helped identify key barriers/facilitators. These findings informed a survey delivered to all AMOS participants. Qualitative data were analysed using an inductive two-step thematic networks technique to identify themes. Quantitative data were summarised using descriptive statistics. A total of 54 AMOS participants and 4 staff participated in the study. Four themes were identified to describe AMOS participant experiences’: 1. affordability; 2. multidisciplinary care; 3. person-centred care; and 4. motivation. Specialised, multidisciplinary and individualised obesity care available through one clinic facilitated self-care and improved health and well-being. Dedicated multidisciplinary obesity clinics are recommended in rural and remote areas.

## 1. Introduction

Obesity is a relapsing chronic and complex condition with genetic, environmental, physiological, psychological, and behavioural determinants [1,2,3]. Obesity is associated with significant comorbidities, particularly type 2 diabetes, cardiovascular disease, osteoarthritis, sleep apnoea and specific cancers [1,3]. For people living with obesity and type 2 diabetes, the risks of diabetes-related complications and adverse cardiovascular and musculoskeletal outcomes are increased [4]. Managing obesity in people with type 2 diabetes through lifestyle modification, screening, and early detection of secondary complications can improve the management of diabetes-related complications [5].

Obesity and diabetes share pathophysiological links; lipid accumulates in non-adipose tissue, and excess adiposity causes hyperglycaemia [5]. Thus, multidisciplinary clinics for obesity and diabetes often share similar goals for weight loss [6]. For the person with type 2 diabetes, it includes managing glycaemia while avoiding weight gain [7]. Evidence supports multidisciplinary person-centred approaches to providing obesity and diabetes care where individuals are active decision makers in their healthcare [8,9,10,11,12]. A combination of nurse practitioners, exercise physiologists, dietitians, and psychologists are considered core elements of these clinics, with the person accessing care at the team’s centre [6,13].

However, despite best practice recommendations promoting multidisciplinary management of people living with obesity and type 2 diabetes, barriers to this level of support exist in rural, regional, and remote areas [9,11,14,15]. These services are often difficult to access or unaffordable for most people for geographical or socioeconomic reasons [15]. In addition, there tends to be a focus on specialty within obesity and diabetes clinics rather than on root causes of problems [16]. Therefore, we must understand the role of multidisciplinary clinic models that support the management of obesity and diabetes in a non-stigmatising manner, particularly in rural, regional, and remote areas.

The Assessment and Management of Obesity and Self-maintenance (AMOS) Clinic was a pilot clinic established to provide team-based multidisciplinary obesity management for people living with type 2 diabetes in rural, regional northwest Tasmania, Australia. The AMOS Clinic included a nurse practitioner lead and a dietitian, psychologist, and physiotherapist, with a referral pathway to bariatric surgery for suitable candidates when required. The focus was to support the person living with obesity to enable informed, individualised strategies to support sustained weight loss. Participants were seen by the nurse practitioner and dietitian at baseline, 6 and 12 weeks and then at three months and a physiotherapist at baseline, 4–6 weeks, 6 months and 12 months. They received a brief phone call at weeks 2, 4, 8, and 10 from a credentialled diabetes educator [17] allocated to the clinic to confirm instructions were understood and to assess any medication side effects and progress. Each AMOS Clinic participant’s medical management was streamlined across all services, with outcomes of each appointment communicated to their general practitioner (GP). Intermittent case discussions occurred between the AMOS clinic and GPs, as required.

Support provided to AMOS Clinic participants was informed by assessments which included measuring readiness and confidence for change, identifying drivers for obesity such as nutritional, psychological, physical, pathological, and medication-based, and a history of weight and weight management. Management was tailored to the participant’s goals and included adjusting glucose-lowering medication and initiating obesity medicines alongside clinical, psychological, and dietary advice. Pivotally, the focus was to support developing self-maintenance skills and the process of attending to and caring for oneself to support long-term success [18].

To inform guidelines, it is essential to understand the experiences of consumers and staff of rural, regional, and remote clinics focused on obesity management in people with type 2 diabetes. Therefore, the current study focuses on understanding the experiences of AMOS Clinic participants and staff and exploring the barriers and facilitators to self-care in obesity and diabetes management.

## 2. Materials and Methods

### 2.1. Study Design

A mixed-method design was utilised to explore the experiences of AMOS Clinic participants and clinic staff. Data collection tools included participant focus groups and surveys and one-on-one clinic staff interviews. Ethical approval was obtained from the Tasmanian Health and Medical Human Research Ethics Committee (H0014324).

### 2.2. Setting

The AMOS Clinic was developed and delivered in the Diabetes Centre in northwest Tasmania, a rural regional area with approximately 115,000 residents. The area has a high prevalence of obesity and diabetes in adults [19,20,21]. It includes the local government area with the highest incidence of diabetes in the state (Waratah/Wynyard 7.9% versus Tasmania 5.8% or National prevalence 5.4%), and more younger adults aged 20 to 29 years live with diabetes than the national average (1.3% versus 0.9%, respectively) [21]. In addition, the majority of northwest Tasmania is in the Index of Relative Socio-economic Disadvantage (IRSD) quintiles 1 and 2—most disadvantaged; the IRDS is an index summarising a range of information about the economic and social conditions of people and households. The mean body mass index (BMI) of people with type 2 diabetes attending local diabetes services (M = 44.5 kg/m^2^) aligns with the severe Obesity III classification; thus, this population is at high-risk of developing additional comorbidities [1,2,3,4,22].

This study examined the experience of those who attended the AMOS Clinic and received the intervention in the AMOS trial [23]. Information about the AMOS trial, including the control group, is registered with the Australian and New Zealand Clinical Trial Registry (ACTRN12622000240741) [24].

### 2.3. Participants

#### 2.3.1. Focus Groups (Participants)

A total of 88 out of 113 people with type 2 diabetes who accessed the AMOS Clinic between 2015 and 2018 were invited by phone by a research officer using a set script to participate in one of two focus group sessions. Information sheets about the focus groups and consent forms were mailed to all AMOS Clinic participants interested in being involved or those not contactable by phone. The 25 AMOS Clinic participants not accounted for either did not consent for follow-up, had out-of-date contact details, or had a notification of death recorded in their digital medical record.

#### 2.3.2. Surveys (Participants)

The same AMOS Clinic participants (*n* = 88) were eligible to receive a paper-based survey by mail based on the themes obtained from the focus group data. Surveys were sent with an information sheet and pre-paid return envelope. The survey’s return implied consent.

#### 2.3.3. Interviews (Clinic Staff)

Seven (70%) staff members involved in delivering services within the AMOS Clinic were emailed an invitation to participate in an interview about their experience with all aspects of the AMOS Clinic. Other staff members had since moved away from the area and were not contactable (*n* = 2) or an investigator (*n* = 1).

### 2.4. Method

#### 2.4.1. Focus Groups

Focus groups were conducted at two locations to increase accessibility for participants. An independent moderator, using a semi-structured approach, and a guide containing six open-ended questions about participant experiences in the AMOS Clinic and elements of self-care guided the discussions. A second external researcher reviewed the questions to clarify the meaning. These questions aimed to draw out opinions of barriers and facilitators to self-care, health, and well-being and included:

1. How was your overall experience with the clinic?
2. What do you see as some of the barriers to successful self-care for you, and what makes it easy?
3. Were there times when you felt more supported in your care to feel hopeful and more confident to act for your own health and well-being.
  - What, if any, were the elements that made you feel less supported or less confident?
4. Did you feel as though you were being included in the decisions that were made about your care?
  - If yes, how?
  - If not, why not, and what would shared decision making look like to you? How would you like to be included in decisions around your care
  - What is the personal and health-related value of the clinic for you?
5. Do you feel that your experience in the clinic had any effect on your own diabetes and obesity management?
6. Do you feel that your experience in the clinic had any effect on any other health outcomes/issues?

The independent focus group moderator was an experienced qualitative researcher, not involved in the design of the AMOS trial or influencing the AMOS Clinic’s development or services. The moderator summarised and reported back to the group following each question to obtain general agreement that the summary represented what was said and that the researcher’s interpretation of key concepts was correct. Both focus group sessions were audiotaped for transcription verbatim by an external transcription service.

#### 2.4.2. Surveys

The research team used key themes identified in the focus group and interview data to inform the development of a 26-item paper-based participant self-report survey. The aim was to survey the broader cohort of intervention participants who had attended the AMOS Clinic to provide further clarity on experiences and opinions. The survey included closed-ended questions to assess the relevance of the focus group themes to the broader AMOS participant cohort and three free-text sections to capture additional participant experiences. A second external researcher reviewed the survey questions to ensure the meaning was clear. Participants were mailed an information sheet explaining the study, the survey, and a reply-paid envelope. They were asked to return the survey within eight weeks.

#### 2.4.3. Interviews

Staff interviews were conducted via telephone or in-person by the same independent moderator involved in the focus groups. A second external researcher reviewed the interview questions to clarify the meaning. The six questions utilised in the semi-structured interviews are below.

1. How was your overall experience with the clinic?
2. What do you see as some of the barriers to successful self-care for your patients, and what makes it easy?
3. Did you feel supported in your role in the clinic?
4. Do you feel that the clinic contributed to your patient’s overall health and well-being?
5. Did you feel as though there was an element of shared decision making during the clinic?
  - If yes, how?
  - If not, why not, and what would shared decision making look like to you? How do you think they would like to be included in decisions around their care?
6. Do you feel that your experience in the clinic had any effect on your own practice around diabetes and obesity management?

**Figure 1:**
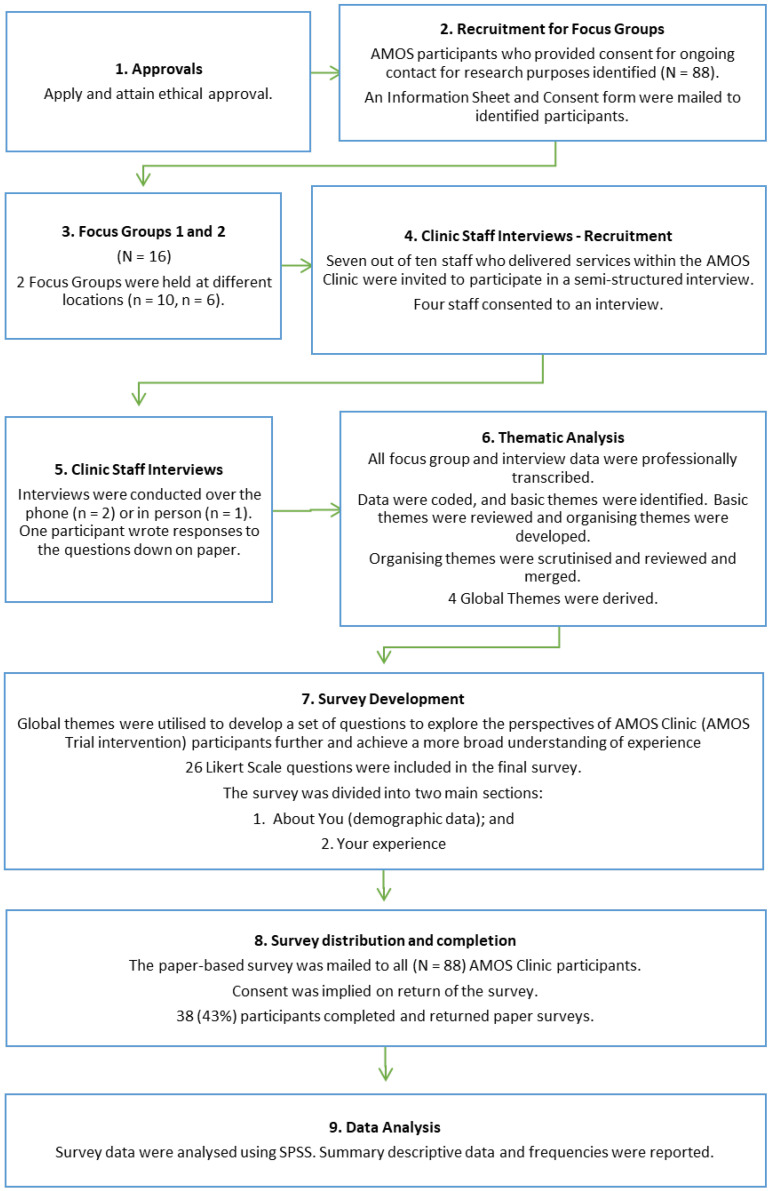
Methods and participants. N: whole group; n: sample.

During the interview, the moderator summarised and reported back to the staff following each question to obtain general agreement that the researcher’s interpretation of key concepts was correct. All phone and face-to-face interviews were audio-recorded and transcribed. One participant wrote their answers to semi-structured questions with phone follow-up for clarification. All phone and face-to-face interviews were audio-recorded and transcribed. Figure 1 shows the methods utilised.

### 2.5. Analysis

#### 2.5.1. Focus Groups and Interviews

Focus group and interview data were combined for thematic analysis. These analyses were performed in two phases based on a qualitative tool described by Attride-Stirling [25]. Two research team members developed the thematic networks by independently coding the transcribed data and identifying key themes. The researchers met three times to compare and scrutinise themes and ascertain the final thematic networks. The second step involved identifying organising themes from these patterns and utilising these themes to identify global, overarching themes within the interview data. Global, overarching themes were then derived from the organising themes.

#### 2.5.2. Surveys

Summary descriptive data and frequencies were reported for survey data, with analysis performed using Microsoft Excel (Microsoft Corporation, Washington, WA, USA) and SPSS V26.0 (IBM, Chicago, IL, USA).

## 3. Results

### 3.1. Demographic Data

#### 3.1.1. Focus Groups (Patient Participants)

A total of 16 (18%) AMOS participants participated in a focus group; one group was held at each AMOS Clinic location—location A (*n* = 10) and location B (*n* = 6). Of these, eight were female, and eight were male, and all participants completed the entire AMOS Clinic program of two years duration. The thematic analysis revealed four global themes, described below (Section 3.2).

#### 3.1.2. Surveys (Patient Participants)

A total of 38 (43%) AMOS participants completed and returned the survey via post. Gender distribution of respondents aligned with the overall AMOS Clinic cohort (36% male and 62% female), and the mean age was 65 years (Range 58–71 years).

#### 3.1.3. Interviews (Clinic Staff)

A total of four AMOS Clinic staff participated in a one-on-one interview about their experiences within the AMOS Clinic. These included one dietitian, two physiotherapists, and one psychologist.

### 3.2. Themes from Focus Groups and Interviews

The themes below represent combined data analysis of patient focus groups and clinical staff interviews.

#### 3.2.1. Theme 1: Affordability

Findings from the focus groups highlighted the importance of health service availability, cost, and choice within the AMOS Clinic to the participants. The majority of participants highlighted the many benefits of receiving care targeting the “whole”, rather than just a specific condition or issue at that time. Participants frequently used terms, such as “holistic” and “all-inclusive”, throughout the focus groups.

*“It was a holistic approach to care”*

(Participant 1);

*“The whole package was brilliant”*

(Participant 13);

*“Definitely recommend it for the support”*

(Participant 2);

*“One of the biggest things is finding out that you are not alone”*

(Participant 13);

*“AMOS is a one stop shop. Convenient, paid for, all done in one visit”*

(Participant 1).

Focus group findings suggest a centralised model where participants faced fewer barriers around access, cost, and time provided a sense of support that encouraged self-care. The majority (63%) of participants felt more comfortable with self-care when they felt their healthcare needs were being met, specifically when things were made easier for them by being all-inclusive or holistic. Many raised the ease of access to mental health services, as affordable and accessible multidisciplinary services can be challenging to access outside the AMOS Clinic.

*“The facilities were great—depression is part of the diabetes story”*

(Participant 2);

*“I joined to learn new exercises and get moving. Getting motivated”*

(Participant 5).

The free, multidisciplinary services offered by the AMOS Clinic significantly influenced all except three focus group participants’ decision to join the program. It was noted that cost is a barrier to self-care for people with obesity.

Focus group participants discussed the costs of healthy eating (specifically, low-calorie food), seeing various specialists and other healthcare providers, travelling to appointments, and the struggles with money as a concession or pension cardholder. Many focus group participants extensively discussed that the main reasons for joining the AMOS Clinic were the subsidised and bulk-billed healthcare services.

*“I joined for similar reason, it could help and will not hurt and its free”*

(Participant 11);

*“There are no free podiatry clinics for diabetes. It is hard on a pensioner”*

(Participant 5);

*“It is hard to eat the right stuff—low calorie food is expensive”*

(Participant 7);

*“Money is the main barrier to self-care outside the clinic”*

(Participant 3).

Feedback from clinical staff also supported these concepts, they suggested that people who do not always have access to these services, such as in rural and remote areas, appreciate subsidised or free healthcare when available.

*“Access to free or low-cost healthcare is absolutely motivating for people”*

(Staff 1);

*“In regional areas, people who are bulk billed always turn up and put in the work”*

(Staff 1).

#### 3.2.2. Theme 2: Multidisciplinary Care

Over half of the participants (69%) expressed that one of the difficulties in managing obesity and diabetes self-care was managing appointments with various specialties. Several participants also discussed the difficulties they had experienced with other external health services, such as changes in staffing, location, and timing of appointments. For many, having a familiar, knowledgeable, multidisciplinary team within a single clinic environment was a valuable approach that made them feel supported and encouraged in their health journey. Several participants indicated that some areas for improvement for ongoing clinics included more consistent availability of psychology and services and online reporting instead of completing paper forms for most consultations. Most (63%) participants indicated they liked being given “something” to do between appointments rather than just information. This preference was most notable with the dietitian service, where participants were hoping for assistance in planning their meals rather than just being provided information about it.

*“There were lots of services… having all of those specialties makes you feel more supported”*

(Participant 12);

*“It was good having other specialties such as dietitian and exercise, but the forms were rotten”*

(Participant 7);

*“I was given pamphlets on meals, but an individualised meal plan would have been more useful”*

(Participant 4).

Similar findings were identified in clinical staff feedback; they highlighted that their patients benefitted from having services accessible at one point of care.

*“I think the patients valued the ‘team’ approach and not having to go to differing sites to access different disciplines”*

(Staff 2).

#### 3.2.3. Theme 3: Person-Centred Care

The individualised style of the AMOS Clinic was described positively throughout the focus group sessions, with the emphasis being placed on the way medications were prescribed and trialed and tailored to future planning for ongoing self-care and weight management.

*“It felt like I was being individually counselled”*

(Participant 2);

*“This was not a one size fits all clinic, which is good. It was tailor-made”*

(Participant 7);

*“I was able to get off insulin which has made it totally worthwhile”*

(Participant 7).

Clinical staff also suggested that patients felt more empowered by being involved in decision-making processes around their ongoing care.

*“Many patients felt more involved in making decisions about their health and healthcare, but some did not”*

(Staff 3).

Three staff members (75%) also noted that the complexity of people with type 2 diabetes needs is better managed in a multidisciplinary environment focusing on tailored care to improve outcomes.

*“…it increased my awareness (and therefore empathy) to the complex nature of these patients’ backgrounds and the multifactorial nature of their presentation”*

(Staff 2);

*“Barriers to self-care include people’s beliefs about themselves and their abilities…”*

(Staff 3);

*“It is important to understand how much time needs to be put in for behaviour change”*

(Staff 1).

More than half (75%) of the focus group participants discussed the AMOS Clinic’s positive influence on their diabetes management when living with obesity. Diabetes-related health improved for the majority of participants as they developed self-care strategies, including diet, exercise and medication regimens that assisted with weight loss.

*“I lost 70 kg…completely off insulin”*

(Participant 1);

*“Insulin makes you hungry, so it is hard to lose weight. So, getting off the insulin really helped”*

(Participant 3).

In particular, some focus group participants indicated that having more control over their diabetes management motivated them to manage other aspects of living with obesity.

#### 3.2.4. Theme 4: Motivation

“Motivation” was a theme for which we derived two main components: sustaining motivation and accountability.

Sustaining motivation was a key issue that all except two focus group participants described as a barrier to ongoing self-care. It included the:

- Motivation to seek healthcare;
- Motivation to lose weight;
- Motivation to communicate;
- Motivation to follow a plan long-term.

Participants further discussed the barrier of sustaining their motivation following the AMOS Clinic when the continuous connection ceased. A few focus group participants (19%) suggested that making a phone call to chat with a health professional would help them reach their goals through ongoing support and a sense of accountability.

*“I found the regular appointments were an incentive to maintain exercise and diet”*

(Participant 5);

*“It would be helpful if they had someone to ring and talk to over the phone anytime for information that is individualised”*

(Participant 15).

Focus group findings suggest the ongoing contact provided motivation and a sense of pride when “checking in” with their healthcare provider. Having someone to check in with regularly was a constant motivating factor reported by many AMOS Clinic participants. Participants described feeling a sense of accountability; they did not want to let their healthcare provider team down by not trying activities or advice to improve their overall health and well-being.

*“It is one-on-one, and it motivates you”*

(Participant 2);

*“AMOS kept me accountable”*

(Participant 13);

*“I need to have goals; I need to have someone check up on me. I tend to leave things if no one is pushing me”*

(Participant 5).

Focus group participants generally felt that the AMOS Clinic was a safe space where they could speak, listen, and ask questions and where they felt their healthcare and support needs were being looked after and made them accountable for their actions outside the clinic.

*“They are all very friendly people… They are nice. That makes you feel at ease”*

(Participant 12);

*“I found the dietitian easy to talk to…”*

(Participant 5)

Further, clinical staff suggested that motivation was influenced by patients being comfortable supporting the feeling of having a safe space and needs-based environment.

*“It’s about finding an environment and set of exercises that the patients feel comfortable with”*

(Staff 2).

### 3.3. Findings from the Survey Aligned with Themes Identified

#### 3.3.1. Theme 1: Affordability

Findings from the patient survey confirmed the importance of the theme ‘affordability’ within the broader participant group. The results show that people with type 2 diabetes attended the AMOS Clinic for the range and affordability of services. In particular, 81% of participants joined the AMOS Clinic for free diabetes services, 59% for free dietitian services, and 33% for free psychology and physiotherapy services (see Table 1). Half of the patients joined for free weight loss support.

#### 3.3.2. Theme 2: Multidisciplinary Care

The patient survey results confirmed that AMOS participants felt well supported in learning self-care (94%), explicitly in collaboration with the AMOS team (95%) (see Table 2). Most patients (78%) agreed that the AMOS Clinic had good communication strategies with their general practitioners. Over half (65%) agreed that multidisciplinary collaboration supported their mental health.

#### 3.3.3. Theme 3: Person-Centred Care

Findings from the survey highlighted that most patients (80%) felt they were equipped to improve their health. Many patients (68%) felt that the collaborative and person-centred approach to care supported successful medication changes. In addition, most patients (75–77%) found the AMOS Clinic’s person-centred approach valuable in supporting their diabetes self-care, and over half (54%) maintained their weight loss (see Table 3).

#### 3.3.4. Theme 4: Motivation

Findings from the patient survey confirmed that the majority (88%) of patients identified that the AMOS Clinic motivated them to engage in their healthcare and supported them in losing weight (80%) (see Table 4). Around half were unsuccessful in previous weight loss attempts (46%) or managing their diabetes (54%), while one-fifth were unsure. Survey results confirmed focus group participant experiences, and almost all participants (97%) would recommend the AMOS Clinic to other people living with obesity and struggling with weight loss.

## 4. Discussion

Our study found that tailoring (individualised) obesity management to goals set by the person living with obesity and type 2 diabetes and having a wider range of affordable services simultaneously accessible in one clinic and delivered by understanding staff are elements for positive consumer experiences promoting motivation.

### 4.1. Theme 1: Affordability

The current study highlights the difficulties of scheduling and attending multiple appointments at different times and places and the associated cost burdens. Navigating multiple appointments created frustration, lack of motivation, and low morale, consistent with the European Practical and Patient-Centred Guidelines for Adult Obesity Management in Primary Care [1].

Although routine care for people with type 2 diabetes in the region included free, public access clinics, these did not include the range of different services offered by AMOS through direct access, such as the physiotherapist and psychologist. However, managing diabetes through weight loss appeared important to participants, as only half joined the AMOS Clinic for the free weight management services. Making the cost of care affordable for those living with chronic conditions such as obesity and type 2 diabetes is essential to help or encourage the development of self-care. For example, according to an analysis of a national, longitudinal survey comparing adults diagnosed with type 2 diabetes to those without diabetes, men are twice as likely to fall into poverty after developing type 2 diabetes [26]. Moreover, 27% of people with diabetes omit care because of the cost [27]. According to Schofield et al., who used a multiple regression model based on the Australian Bureau of Statistics surveys of disability, aging, and cares, 38% of those aged 45 to 64-year with diabetes retired early and had incomes 88% lower than their employed colleagues [28]. Thus, earlier intervention may improve their living standards and delay obesity and type 2 diabetes-related complications. Making obesity healthcare affordable and convenient promotes engagement with health services, which nurtures self-care.

AMOS Clinic participants in this study regarded the clinic as a convenient ‘one stop shop’, which benefited them by providing improved accessibility, facilitating ongoing appointment attendance and improving their overall knowledge about their health. The role that multidisciplinary services play, indirectly and directly improving patient outcomes through improved experience and satisfaction, is well established. Evidence suggests multidisciplinary clinics have been well received by patients regardless of their structure, that is, the health professional makeup of the team [29]. In contrast, the literature suggests patient perceptions of traditional sequential models of care where patients are referred to a series of alternate clinicians is poorer physician communication, unproductive use of the patient time, increased financial burden, and a higher chance of misdiagnosis and mistreatment, leading to decreased patient satisfaction [29].

Our findings emphasised the value of affordable specialised psychology access, promoting self-care strategies for managing mental health. Obesity and its comorbidities come with a significant psychosocial burden that can impact a range of psychosocial functioning areas [30]. In particular, the relationship between excess body weight and depression [31] and anxiety disorders are more common among people presenting for bariatric surgery [32]. Moreover, a systematic literature review including 60 studies and 300,000 participants identified an association between adverse life experiences and the development of obesity or a binge-eating disorder [33]. These were often traumatic experiences; exposure to sexual abuse in childhood was the highest risk factor for developing obesity in adulthood and post-traumatic stress disorder in adults [33].

Several AMOS Clinic participants highlighted past difficulties experienced trying to access mental health services locally, including time, cost, and availability perspectives. AMOS Clinic participants perceived the psychology support as helpful and accessible. The inclusion of psychological support in specialised and multidisciplinary clinics such as AMOS could improve outcomes for people living with obesity by addressing the emotional consequences of living with obesity [34].

### 4.2. Theme 2: Multidisciplinary Care

Findings from the current study align with international (European) guidelines that promote specialised and multidisciplinary support to facilitate obesity management, highlighting a deficit in previous obesity management strategies in the local area. European obesity management guidelines indicate that a multidisciplinary team structure consisting of a medical specialist, dietitian, physical activity specialist, mental health specialist, nursing staff, and general practitioner is a more efficient model than separate single access services [1]. Conversely, the current Australian Clinical Practice guidelines are outdated and require a more informed approach.

Holistic, comprehensive, multidisciplinary approaches to obesity management have been on the public health agenda globally [1,3,13,20,35]. Research has identified gaps in resourcing, most notably in rural, regional, and remote areas, to provide a structured approach for obesity management, allowing more authentic consumer/practitioner engagement [20].

Ogden et al. undertook an exploratory qualitative study to understand the lived experience of those who are overweight and obese and self-perceived barriers to access and engagement in interventions [36]. The authors identified that health professionals were a component of the ‘impediments’ theme; health professionals could either be a positive reinforcing factor facilitating access and engagement or a negative factor impeding access [36]. In the AMOS model, the survey findings suggest that the individual living with obesity was managed in a supportive clinical environment. The AMOS model provided a service with staff understanding of obesity and chronic conditions management who were empathetic towards supporting the participant to reach their goals.

The issues contributing to and impacting healthcare providers’ skills and willingness to engage in risk assessment and management of people living with obesity are multifactorial and complex [37]. Despite the availability of many regimens for treating obesity, such as clinical models, medication, physical rehabilitation, and surgical interventions [38], there remain gaps in healthcare providers’ knowledge and understanding of how best to address obesity and its management. Numerous studies have suggested that healthcare providers feel ill-equipped to assist people with weight management and lack training in managing obesity [39,40,41,42]. Multidisciplinary obesity clinics such as the AMOS model develop staff skills in assessment and person-centred treatment modalities.

### 4.3. Theme 3: Person-Centred Care

Our findings suggest that providing an individualised, tailored approach to managing obesity that supports success by achieving personal goals was vitally important to AMOS study participants. The person-centred approach within the AMOS model provided participants with the relevant resources required to develop individual nutritional, psychological, and physical self-care strategies and meet their medical needs, as often these factors overlap, by a collaborative, empathetic team.

Ogden et al. suggest that through understanding the individual’s physical surroundings and everyday experiences, their lifeworld can identify which lifeworld dimensions are more significant in their own lived experiences of obesity, providing essential insights for weight loss intervention providers [36]. Our study also identified that individual experiences living with obesity differ. When the participant perceived they were at the centre of the model of care delivered and had some control over decision making, they were more likely to have successful outcomes and obesity management strategies.

Weight loss maintenance is hindered by a complex interaction of environmental, biological, behavioural, and emotional health factors, including self-confidence and medications used to manage comorbidities [43,44]. The AMOS Clinic provided a service to assess, treat, and provide self-care advice to work towards managing these aspects collaboratively. For example, hormonal imbalances and medication causes of weight gain were individually managed in alignment with dietary and psychosocial advice to set the AMOS participant up for success in weight loss. It included lowering insulin doses in alignment with caloric restrictions to prevent hypoglycaemia or treating those with leptin resistance with medications to boost anorexigenic effects while they worked towards developing self-maintenance strategies. Understanding and appreciating the individual’s ‘story’ was paramount in AMOS Clinic assessments, interlinked with the ability to shift focus from one service to another as required to seamlessly promote motivation, evidenced by the participants’ experiences.

### 4.4. Theme 4: Motivation

People living with obesity often face complex issues, including reduced quality of life, reduced psychosocial well-being and self-esteem, and increased personal vulnerability [45], which may impede motivation to participate in self-care activities. Participants in our study suggested that self-care could be impeded in several ways that align with literature, being motivated to seek healthcare when needed [46], motivation to lose weight [1], motivation to communicate [47], and motivation to follow long term health plans [1]. Ogden et al. [36] identified three overarching themes in their qualitative study investigating lived experiences of obesity: ‘complexity and battle’, ‘impediments’, and ‘positive re-orientation’. These are consistent with the four themes identified in this study through a strong focus on self, support, and motivation. A key focus of the AMOS Clinic was supporting participants to develop self-maintenance skills to assist them in understanding triggers and motivators for eating habits, weight gain and loss, stress, and physical activity and managing those working against their weight loss goals.

Surrow et al. [46] identified that health issues and feeling more comfortable with their bodies motivate people who live with obesity to lose weight. Similarly, Durrer et al. [1] considered the approaches currently used in obesity management and noted that motivation is essential for adherence to treatment and readiness to change. The AMOS Clinic provided an opportunity to draw on motivating moments and aligned with the authors’ conclusion that the opportunities for weight loss lie in creating habits, routines, and structure of everyday life activities to facilitate a healthier lifestyle.

The AMOS findings suggest that ensuring continuous connections with people living with obesity and diabetes, despite the timeframe often being 3 months, is an important step for building mutual trust, encouragement, and confidence. The AMOS Clinic participants valued goal setting and feeling safe and supported in the AMOS Clinic, suggesting that current barriers exist in intrinsic motivation. The lived experience of obesity, as explored by Ogden et al. [36], also suggested that empowerment and motivation play a key role in the positive re-orientation of behaviour, contributing to successful weight loss. As a major facilitator for self-care, accountability provided participants with a focus and drive to achieve their weight goals and maintain weight loss.

People with obesity and diabetes face many challenges, including achieving and sustaining weight loss due to several important factors or concurrent diabetes-related health problems. Physical activity can be limited by physiological conditions, such as neuropathy, foot ulcers or heart disease [47], and weight gain can be attributed to certain diabetes medications such as insulin [48]. Previous studies have suggested that weight loss for people with diabetes, controlled by diet alone, plateaus at 6 months and is maintained for 12 months provided ongoing (monthly) contact with healthcare professionals exists [49]. The importance of ongoing engagement with health professionals aligns with the current study’s findings, where ongoing, familiar support was described as a motivating factor for various health-seeking behaviours. These are important considerations in rural, regional, and remote areas where service deficits exist.

### 4.5. Rural, Regional and Remote Models of Care

The AMOS Clinic model aligns with the *National Rural Health Alliance’s* strategic advice, which indicates that the *National Rural Health Strategy* should acknowledge that rural and remote communities are different from metropolitan communities and promote alternate innovative models of care [50]. The Scottish government performed an international review of models of multidisciplinary teams working in rural primary care using structured interviews with 21 rural health provider informants from 8 countries [51]. The outcomes suggest that issues in rural care are complex, as are the solutions; however, culture and context are important, and the most challenging environments often have the most innovation [51].

Our research informs local, national, and international models of care for obesity management, particularly in high-risk type 2 diabetes populations and people in rural, regional, and remote areas. Simultaneously, nationally and internationally, governments are seeking a national approach to policy, planning, design, and delivery of health services to rural and remote communities [50,51,52,53]. Given this, knowledge about care models that support person-centred weight-loss by supporting the development of self-maintenance strategies must be improved. These models may benefit by comprising all approaches to weight loss, including bariatric surgery or other non-surgical lifestyle management, to meet the diversity of people’s needs. This research provides evidence that supports and guides local and national resourcing and planning.

## 5. Conclusions

The medical complexity of people living with obesity and diabetes are barriers, which impede weight loss progress. People living with obesity navigate many costly services while maintaining motivation and focusing on improving their health and lifestyle behaviours. This study demonstrates that obesity management, supported by a specialised, multidisciplinary clinic focused on individualising care according to personal needs in a safe environment, is valued by people living with obesity. However, ongoing support to participate in activities for weight loss, self-care, and well-being remain challenging in areas where resources are limited. This research informs the development or redesign of local, national, and international models of care for obesity management, particularly in high-risk type 2 diabetes populations and those in rural, regional, and remote areas.

## Figures and Tables

**Table 1:** Patient survey results—Theme 1: affordability.

Statement	Strongly Agree	Agree	Disagree	Strongly Disagree	Not Sure	Missing
I joined AMOS for the free weight management services	9 (27%)	8 (24%)	11 (33%)	2 (6%)	3 (9%)	5
I joined AMOS for the free specialist diabetes services	13 (39%)	14 (42%)	5 (15%)	1 (3%)	0	5
I joined AMOS for the free psychology services	6 (18%)	5 (15%)	15 (44%)	4 (12%)	4 (12%)	4
I joined AMOS for the free physiotherapy services	5 (15%)	6 (18%)	15 (44%)	4 (12%)	4 (12%)	4
I joined AMOS for the free dietitian services	8 (24%)	12 (35%)	8 (24%)	4 (12%)	2 (6%)	4

**Table 2:** Patient survey results—Theme 2: specialised multidisciplinary care.

Statement	Strongly Agree	Agree	Disagree	Strongly Disagree	Not Sure	Missing
AMOS supported me to learn to take care of my body	14 (40%)	19 (54%)	0	0	2 (6%)	2
AMOS provided a supportive team	16 (46%)	17 (49%)	0	0	2 (6%)	0
My mental health was looked after by the AMOS team (or clinic)	11 (31%)	12 (34%)	3 (9%)	0	9 (26%)	2
I felt nervous or anxious about not having support after the AMOS Clinic	5 (16%)	2 (6%)	13 (41%)	3 (9%)	9 (28%)	5
There was good communication between AMOS and my GP about diagnostic tests	11 (33%)	15 (45%)	2 (6%)	1 (3%)	4 (12%)	4

**Table 3:** Patient survey results—Theme 3: person-centred care.

Statement	Strongly Agree	Agree	Disagree	Strongly Disagree	Not Sure	Missing
Because of my experience with the AMOS Clinic, I feel that I am able to make the right changes to keep improving my health	6 (18%)	21 (62%)	1 (3%)	1 (3%)	5 (15%)	3
I felt safe in the AMOS Clinic	16 (47%)	17 (50%)	0	0	1 (3%)	2
I have been able to maintain any weight loss since the AMOS Clinic	7 (21%)	11 (32%)	10 (29%)	1 (3%)	5 (15%)	3
The AMOS Clinic helped me get my diabetes under control	16 (46%)	10 (29%)	1 (3%)	0	8 (23%)	3
I have been able to keep my diabetes under control since the AMOS Clinic	9 (26%)	18 (51%)	2 (6%)	2 (6%)	4 (11%)	3
Because of my experience with the AMOS Clinic, my medication changes were successful	13 (37%)	11 (31%)	2 (6%)	1 (3%)	8 (23%)	3

**Table 4:** Patient survey results—Theme 4: exploring motivation.

Statement	Strongly Agree	Agree	Disagree	Strongly Disagree	Not Sure	Missing
AMOS motivated me to look after my overall health and well-being	11 (31%)	20 (57%)	2 (6%)	0	2 (6%)	3
My GP was supportive of me being involved in the AMOS Clinic	13 (38%)	13 (38%)	3 (9%)	2 (6%)	3 (9%)	4
The AMOS Clinic helped me lose weight	13 (37%)	15 (43%)	3 (9%)	0	4 (11%)	3
I would recommend the AMOS Clinic to other people who struggle with weight issues	22 (63%)	12 (34%)	0	0	1 (3%)	3
I joined AMOS because I had no success managing my diabetes on my own in the past	5 (14%)	16 (46%)	8 (23%)	0	6 (17%)	3
I joined AMOS because I have had no weight loss success on my own in the past	9 (26%)	7 (20%)	12 (34%)	0	7 (20%)	3

## Data Availability

The data presented in this study are available on request from the corresponding author. The data are not publicly available due to ethics permission around privacy.
